# Gas-Phase Reactivity Studies of Small Molybdenum Cluster Ions with Dimethyl Disulfide

**DOI:** 10.1007/s11244-017-0864-3

**Published:** 2017-11-27

**Authors:** Aristeidis Baloglou, Milan Ončák, Christian van der Linde, Martin K. Beyer

**Affiliations:** Institut für Ionenphysik und Angewandte Physik, Universität Innsbruck, Technikerstraße 25, 6020 Innsbruck, Austria

**Keywords:** Gas-phase ion chemistry, Molybdenum cluster, Dimethyl disulfide, Sulfidation

## Abstract

Molybdenum sulfide is a potent hydrogen evolution catalyst, and is discussed as a replacement of platinum in large-scale electrochemical hydrogen production. To learn more about the elementary steps of MoS_2_ production by sputtering in the presence of dimethyl disulfide (DMDS), the reactions of Mo_x_^+^, x = 1–3, with DMDS are studied by Fourier transform ion cyclotron resonance mass spectrometry and density functional theory calculations. A rich variety of products composed of molybdenum, sulfur, carbon and hydrogen was observed. Mo_x_S_y_^+^ species are formed in the first reaction step, together with products containing carbon and hydrogen. The calculations indicate that the strong Mo-S bonds are formed preferentially, followed by Mo–C bonds. Hydrogen is exclusively bound to carbon atoms, i.e. no insertion of a molybdenum atom into a C–H bond is observed. The reactions are efficient and highly exothermic, explaining the rich chemistry observed in the experiment.

**Electronic supplementary material:**

The online version of this article (10.1007/s11244-017-0864-3) contains supplementary material, which is available to authorized users.

## Introduction

The environmentally benign generation of hydrogen via water electrolysis plays a key role in a future hydrogen economy. The most efficient catalyst for the hydrogen evolution reaction (HER) is platinum, but the available supplies of this element are not sufficient to meet the expected demand for large-scale hydrogen production. Molybdenum sulfide (MoS_2_) based catalysts have the potential to replace platinum as a HER catalyst in electrochemical water splitting [1–7]. The material is also discussed as a catalyst for methanol synthesis from carbon dioxide and molecular hydrogen [8, 9]. A better understanding of the reaction mechanisms contributes to the targeted optimization of the catalysts.

In industry, MoS_2_-based catalysts are widely used e.g. in hydrotreating of refined petroleum products [10–13], as well as a lubricant [14]. For the sulfidation of molybdenum, various sulfur agents may be used, such as hydrogen sulfide (H_2_S) or organosulfide compounds like dimethyl disulfide (DMDS) [15]. In a previous study the tendency of carbon to become incorporated into MoS_2_ nanoparticles synthesized from a metallic Mo precursor was investigated [16], with the main focus lying on the possible incorporation of carbon during the sulfidation with organosulfides, namely DMDS and dimethyl sulfide (DMS). Scanning tunneling microscopy showed that the size and shape of the resulting MoS_2_ clusters were affected by the choice of the sulfiding agent [16]. X-ray photoelectron spectroscopy revealed that when H_2_S or DMDS was used as a sulfur agent, no carbon was incorporated inside or on the surface of the nanoclusters [16]. However, when using DMS instead, incomplete sulfidation led to some carbon species on poorly crystalline MoS_2_ phases of non-carbide nature, which disappeared upon annealing [16]. The deficiency of carbon after sulfidation could be explained by DMDS decomposition into DMS and H_2_S in the presence of H_2_, as reported by Texier et al. [15]. In the absence of hydrogen, direct extraction of sulfur from DMDS with the formation of DMS as a byproduct has the same effect. DFT calculations were used to investigate the stability of different carbon species (C, CH, CH_2_) incorporated into MoS_2_ [16]. The calculations showed that, both for S-edge and Mo-edge, the stability of the substituted species increases from C via CH to CH_2_ [16]. Further quantum chemical studies addressed Mo_10_S_*x*_ species as model systems of planar molybdenum sulfide clusters [17], determined the most stable composition of Mo_*n*_S_*m*_ species [18], or investigated hydrogen evolution from water mediated by Mo_3_S_4_^−^ [19].

In the gas phase, MoX_*n*_^+^, X=O, S, *n* = 1–3, have been studied with a combination of experiment and theory [20] by Schwarz and co-workers. Very recently, the reactions of molybdenum monoxide and dioxide cations with ethanol were addressed by the same group [21, 22]. Structure and thermochemistry of MoS_x_^+^ was addressed by Schwarz, Armentrout and co-workers using guided ion beam experiments and Fourier transform ion cyclotron resonance (FT-ICR) mass spectrometry in combination with quantum chemistry [23]. Guided ion beam experiments also yielded a wealth of thermochemical data on MoO_x_^+^ species interacting with CO and CO_2_ [24, 25]. Fielicke and co-workers studied the effect of molybdenum doping on the reactivity of metal and metal oxide clusters [26, 27]. Bohme and co-workers established that a gas-phase Mo(C_60_)_4_^+^ complex can be formed in a flow reactor [28]. The same group studied the reactions of Mo^+^ with heavy water [29], carbon disulfide [30], O_2_ [31] and N_2_O [32] as part of a large-scale investigations of periodic trends. Although lying in the thermodynamic window, Mo^+^ does not catalyze the reduction of N_2_O by CO [33].

In this work, the formation of Mo_x_S_y_^+^ clusters from Mo_*n*_^+^ and DMDS is studied to understand the formation of these clusters. For this task an FT-ICR mass spectrometer is used, as it is an excellent tool for the examination of ion–molecule reactions in the gas-phase [34–36], including catalytic cycles [37–39]. Experiments are complemented by quantum chemical calculations of reactant and product species to test whether the observed product ions are energetically accessible.

## Experimental Section and Calculations

The reactivity measurements are performed on a modified Bruker/Spectrospin CMS47X FT-ICR mass spectrometer, equipped with a 4.7 T superconducting magnet and an Apex III data station [40, 41]. The ions are generated in a laser vaporization source [42–44], which is combined with supersonic expansion. The molybdenum vaporization is achieved by focusing the second harmonic (532 nm) of a 5 ns pulsed Nd:YAG laser with 5–120 mJ per pulse, depending on the desired cluster species. Due to the high boiling point of molybdenum of 4885 K [45], relatively high laser pulse energies had to be used. The produced plasma is rapidly thermalized by a precisely timed helium gas pulse of typically 30 µs, which propagates perpendicularly to the lasers beam path. The gas pulse is produced in the source chamber (10^−6^–10^−4^ mbar) by a piezoelectric valve with a backing pressure of about 20 bar. During thermalization cluster formation occurs. The produced (cluster-)ions are entrained by the helium gas carrier and are accelerated and guided by several electrostatic lenses towards the ultrahigh vacuum (UHV) region, with a pressure in the lower 10^−10^ mbar range without reaction gas. The source gas pulses have no measurable effect on the UHV pressure. The ICR cell is located in the center of the superconducting magnet, where the ions are trapped, mass selected, reacted and detected.

DMDS vapor is introduced into the UHV via a leak valve at a constant background pressure in the range of (6–8) × 10^−9^ mbar. The liquid sample is degassed by several freeze–pump–thaw cycles. Broadband and single frequency resonant excitation are used to isolate the species of interest. Stable ion signal conditions were obtained for Mo_*n*_^+^, *n* = 1–3, and their reactions with DMDS were monitored by taking mass spectra at delays from 0.0 to 25.0 s. Since ion accumulation in the cell takes 2 s, some reaction products are already present at 0 s reaction delay. Typically, 20 experiment cycles are run at each reaction delay for signal averaging. Kinetic analysis is performed assuming pseudo-first order kinetics. Due to the large number of intermediates and products, only peaks that exceeded a relative intensity of 2% at any time during the first 4 s were taken into account for data evaluation, to keep the fits manageable. After calibration of the measured pressures and correcting for ion-gauge sensitivity [46], pressure-independent rate coefficients are obtained as described in detail before [47]. The uncertainty in pressure measurement is the dominant contribution to the experimental error of the rate coefficients, which is typically 25–30%. Branching ratios are more accurate, since they depend only on the error of the pseudo-first order rate coefficients. Their error depends on the overall signal-to-noise level of the experiment and is estimated to ± 2–3% in the present study.

Since natural molybdenum has a broad isotopic distribution, isotopically enriched ^92^Mo (STB Isotope Germany GmbH) was used. Metallic powder of 700 mg ^92^Mo with ≥ 99.9% enrichment was pressed and sintered into a solid target disk. DMDS (≥ 99.0%, Sigma-Aldrich) was used without further purification.

Structure and energetics of relevant species along the reaction path were calculated using density functional theory (DFT), employing the M06-L functional [48] along with the def2TZVP basis set [49]. For species containing molybdenum atoms, various possible conformations were used as a starting point for optimization, with spin multiplicities up to heptuplet. In total, more than 450 initial optimization points of different structure or spin multiplicity were considered for 26 non-trivial molybdenum-containing species studied in the present work. Only the most stable structures are dealt further. The stability of the wavefunction was tested in every local minimum. Frequency calculations were performed to verify the absence of imaginary frequencies, zero-point energy is included in all reported energies. No scaling was applied to the calculated frequencies.

See Supporting Information (SI) for the complete parameter matrices containing all calculated absolute reaction rates, as well as optimized structures and benchmarking of the computational method with respect to other DFT functionals and higher-level methods.

## Results and Discussion

### Monomer Mo^+^

**Fig. 1:**
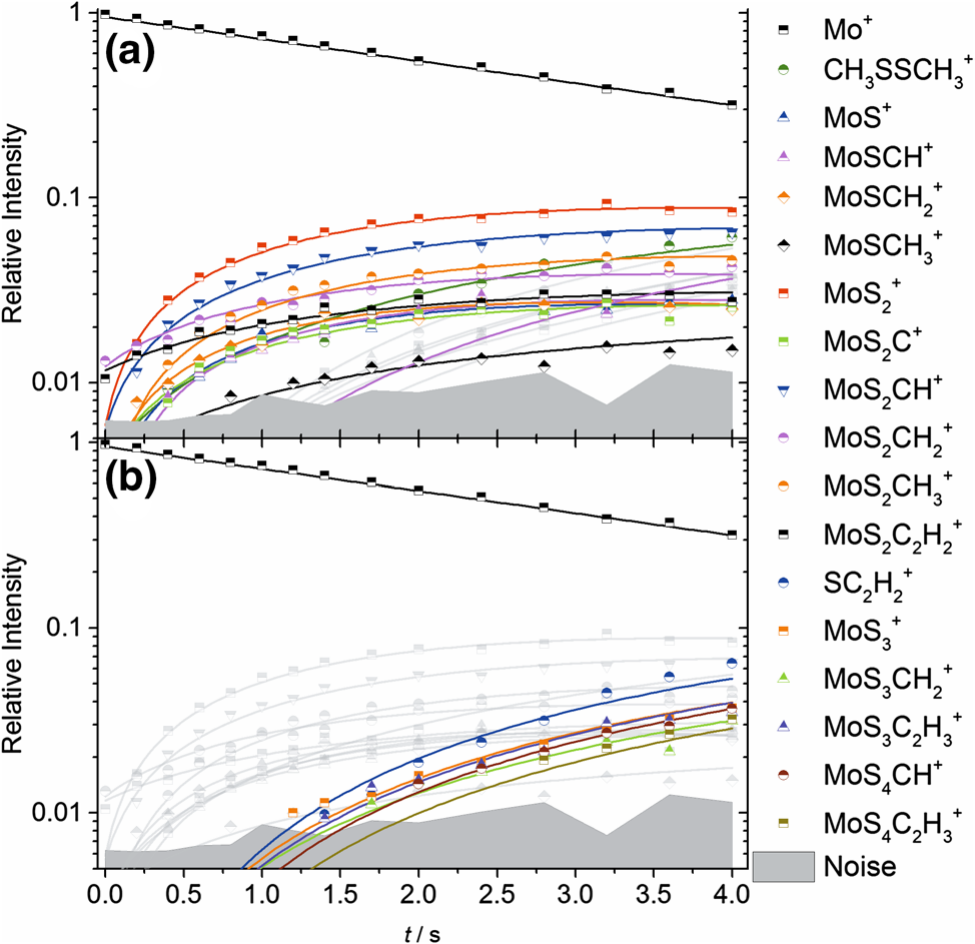
Kinetic fit of Mo^+^ with DMDS during first 4 s of the reaction at *p*_DMDS_ = 5.9(2) × 10^−9^ mbar. The graph has been split into **a** primary and **b** secondary products

Due to the vast variety of products being formed in competing reactions, the reaction kinetics were fitted only for the first 4.0 s. The kinetic diagram showing the time dependent relative intensities of the parent ion (Mo^+^) and its product ions is depicted in Fig. 1. In total 39 reaction channels were used to describe the time profile of the measured data. Since the precursors of secondary and higher order products cannot be unambiguously identified on the basis of the kinetics data, the main focus here lies on the first reaction step. The reactions and rate coefficients for the first reaction step are listed in Table 1. These results are robust with respect to the sequence of secondary and higher-order reactions chosen for the fit. The reaction energies were calculated only for products that, for stoichiometric reasons, can be formed from Mo^+^ and one DMDS molecule.

**Table 1:** Rate coefficients and reaction energies for the first step of the suggested reaction sequence of Mo^+^ with DMDS during the first 4.0 s

	Products	*k*_abs_/10^−10^ cm^3^ s^−1^	Δ*E*/eV	Branching ratio (%)
(1.1)	^4^MoS^+^ + CH_3_SCH_3_	1.6	− 1.30	7
(1.2)	^5^MoSCH^+^ + CH_4_ + SH	1.4	0.01	6
(1.3)	^4^MoSCH_2_^+^ + CH_3_SH	1.6	− 1.58	7
(1.4)	^3^MoSCH_3_^+^ + CH_3_S	1.0	− 1.02	5
(1.5)	^2^MoS_2_^+^ + C_2_H_6_	5.2	− 3.40	24
	^2^MoS_2_^+^ + 2 CH_3_		0.34	
(1.6)	^4^MoS_2_C^+^ + CH_4_ + H_2_	1.1	− 1.99	5
(1.7)	^1^MoS_2_CH^+^ + CH_4_ + H	4.3	− 0.80	20
	^1^MoS_2_CH^+^ + CH_3_ + H_2_		− 0.63	
(1.8)	^2^MoS_2_CH_2+_ + CH_4_	1.2	− 3.95	5
(1.9)	^1^MoS_2_CH_3_^+^ + CH_3_	2.6	− 2.01	12
(1.10)	^2^MoS_2_C_2_H_2_ + 2H_2_	1.8	− 1.87	8

The uncertainty for the rate coefficients has been estimated to 27%. For the branching ratios, an error of ± 2% is assumed. Reaction energies Δ*E* were calculated at the M06-L/def2TZVP level; composition of neutral product molecules and spin multiplicities for Mo-containing species (denoted as superscripts) were deduced from quantum chemical calculations

At the early stage of the reaction, the primary products MoS_2_^+^, MoS_2_CH^+^ and MoS_2_CH_3_ are the most dominant, with branching ratios of 24, 20 and 12%, respectively (see reactions 1.5, 1.7 and 1.9 in Table 1). The remaining products are produced with branching ratios of 5–8%. In the kinetic model used for the fit, the DMDS cation is formed by charge transfer from higher order products such as MoS^+^, MoSCH_2_^+^ and MoSCH_3_^+^ (see Table S1). The data at longer times suggest that charge transfer products, in particular C_2_H_6_S_2_^+^ and C_2_H_5_S^+^, are efficiently formed throughout the reaction sequence, most likely from multiple precursors.

**Fig. 2:**
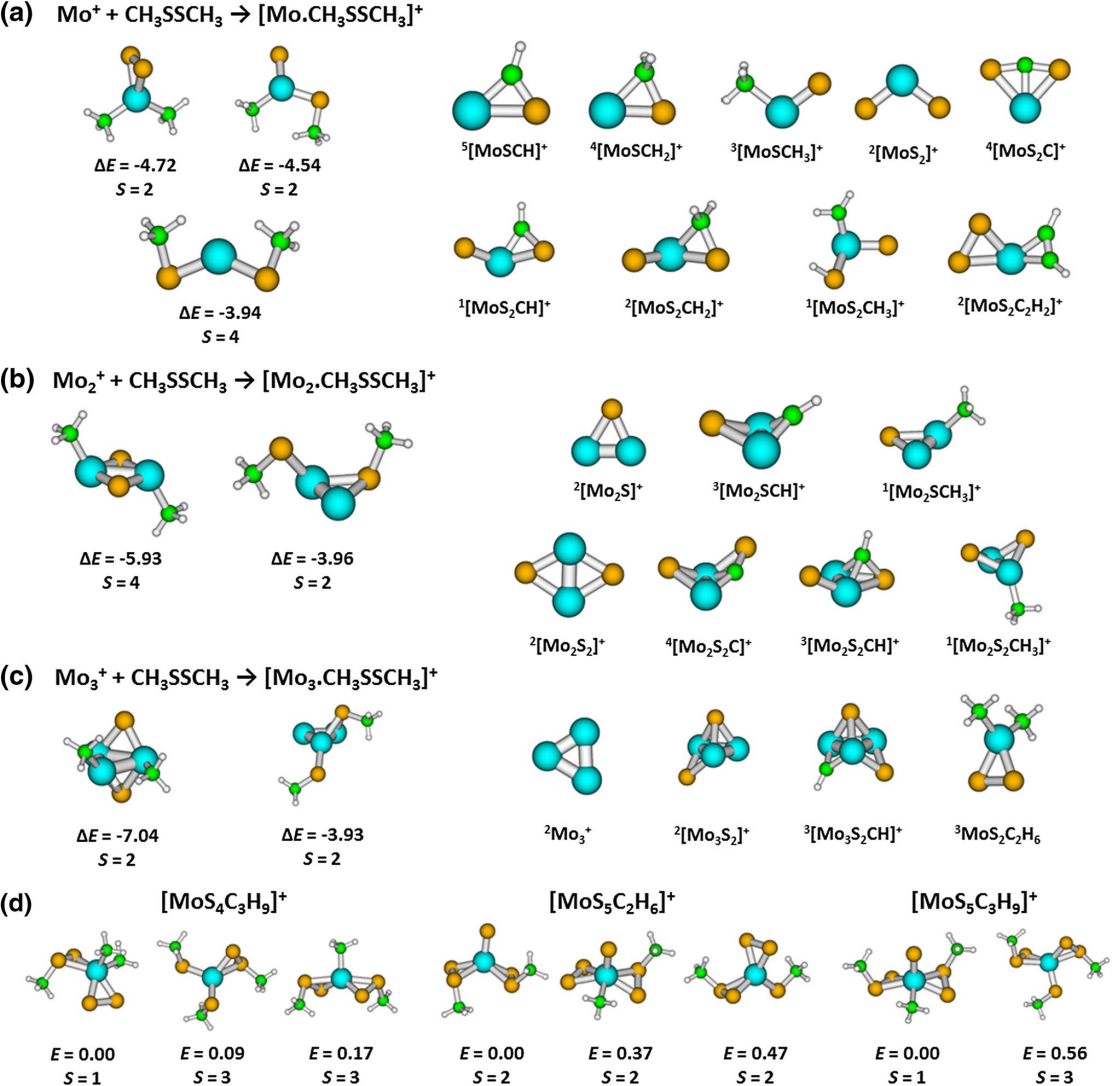
**a**–**c** Left: initial complexes obtained after the reaction of Mo_*n*_^+^ and CH_3_SSCH_3_, *n* = 1–3, along with energy Δ*E* (in eV) relative to the separated reactants. Right: ions obtained after dissociation of the initial complex as included in Tables 1, 2, 3 as well as neutral MoS_2_C_2_H_6_. **d** Selected ions arising during secondary reactions and their relative energy *E* (in eV). All structures were optimized at the M06-L/def2TZVP level of theory, spin multiplicities *S* are given for each ion. Color code: molybdenum—blue; sulfur—orange; carbon—green; hydrogen—white

Structures of possible association and reaction products are shown in Fig. 2a. When Mo^+^ and CH_3_SSCH_3_ interact, there is up to 4.7 eV of energy released (as calculated at the M06-L/def2TZVP level of theory). This energy is redistributed among the internal degrees of freedom of the cluster and leads to dissociation of various bonds, leading to the observed reaction products. The high reaction energy also explains why the association product is not observed among the reactions products, since a large number of strongly exothermic reaction channels make radiative association [50] impossible. The majority of dissociation reactions are calculated to be substantially exothermic; however, the fastest reactions are not necessarily the most exothermic ones. This might be rationalized by kinetic effects, or entropic barriers might prevent the ion from reorganization. At the same time, different neutral reaction products might be formed than the ones assumed in Table 1, e.g. 2 CH_3_^·^ instead of C_2_H_6_, leading to less exothermic reactions. There is one reaction that is slightly endothermic (reaction 1.2, Δ*E* = 0.01) where we might expect that the excess energy is supplied by the kinetic energy of the trapped ions.

**Table 2:** Rate coefficients for the suggested reaction sequence of Mo_2_^+^ with DMDS during the first 4.0 s of the reaction

	Products	*k*_abs_/10^−10^ cm^3^ s^−1^	Δ*E*/eV	Branching ratio (%)
(2.1)	^2^Mo_2_S^+^ + CH_3_SCH_3_	2.7	− 2.10	23
(2.2)	^3^Mo_2_SCH^+^ + CH_4_ + SH	1.2	− 0.83	10
(2.3)	^1^Mo_2_SCH_3_^+^ CH_3_S	0.8	− 1.39	7
(2.4)	^2^Mo_2_S_2_^+^ + C_2_H_6_	1.7	− 4.56	14
	^2^Mo_2_S_2_^+^ + 2 CH_3_		− 0.81	
(2.5)	^4^Mo_2_S_2_C^+^ + CH_4_ + H_2_	1.2	− 2.83	10
(2.6)	^3^Mo_2_S_2_CH^+^ + CH_4_ + H	2.6	− 1.23	22
	^3^Mo_2_S_2_CH^+^ + CH_3_ + H_2_		− 1.06	
(2.7)	^1^Mo_2_S_2_CH_3_^+^ + CH_3_	1.4	− 3.53	13

The uncertainty for the rate coefficients has been estimated to 29%. For the branching ratios, an error of ± 2% is assumed. Reaction energies Δ*E* were calculated at the M06-L/def2TZVP level; composition of neutral product molecules and spin multiplicities for Mo-containing species (denoted as superscripts) were deduced from quantum chemical calculations

**Table 3:** Rate coefficients for the suggested reaction sequence of Mo_3_^+^ with DMDS during the first 4.0 s of the reaction

	Products	*k*_abs_/10^−10^ cm^3^ s^−1^	Δ*E*/eV	Branching ratio (%)
(3.1)	^2^Mo_2_^+^ + ^3^MoS_2_C_2_H_6_	5.3	− 0.98	47
(3.2)	^2^Mo_3_S_2_^+^ + C_2_H_6_	2.0	− 6.23	18
	^2^Mo_3_S_2_^+^ + 2 CH_3_		− 2.48	
(3.3)	^3^Mo_3_S_2_CH^+^ + CH_4_ + H	3.7	− 4.19	35

The uncertainty for the rate coefficients has been estimated to 29%. For the branching ratios, an error of ± 3% is assumed. Reaction energies Δ*E* were calculated at the M06-L/def2TZVP level; composition of neutral product molecules and spin multiplicities for Mo-containing species (denoted as superscripts) were deduced from quantum chemical calculations

From a structural perspective, molybdenum tends to form as many bonds with heavy atoms as possible, as shown also in previous studies on charged and neutral Mo_*x*_S_*y*_ clusters [18, 19, 51]. Already for the association product, the structure containing two Mo–C and two Mo–S bonds was found to be the most stable one (see Fig. 2a). This is also true for ions produced in subsequent reactions that form as many bonds of molybdenum with heavy atoms as possible (see below). There were, on the other hand, no structures with the metal center inserting into C–H bonds found among the most stable isomers. This is perfectly in line with studies by Armentrout on the activation of alkanes by Mo^+^, where only endothermic reactions were observed [52–54].

**Fig. 3:**
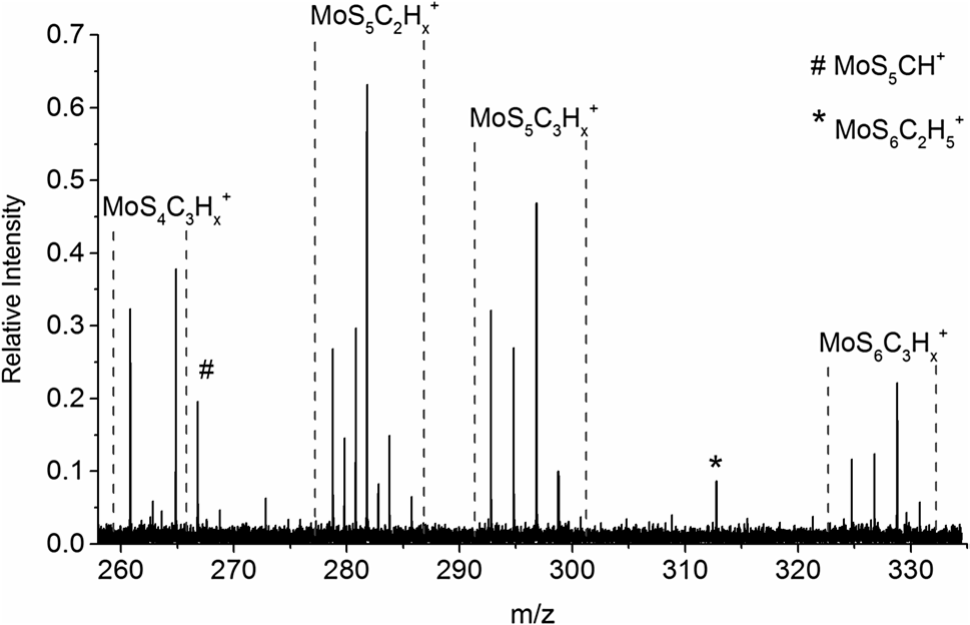
Mass spectrum of Mo^+^ with DMDS after a reaction delay of 20.0 s showing the final products of the reaction (*p*_DMDS_ = 5.9(2) × 10^−9^ mbar). Intensities are given relative to the most abundant product SC_2_H_5_^+^

A qualitative analysis of the mass spectra for reaction delay until *t*_*r*_ = 20.0 s reveals that these products continue to react with DMDS until the final products are formed. The molybdenum containing products are shown in Fig. 3, where the most dominant ones are MoS_5_C_2_H_6_^+^, MoS_4_C_3_H_9_^+^ and MoS_5_C_3_H_9_^+^. It is worth mentioning that neither pure MoS_x_^+^ nor MoC_x_H_y_^+^ species were present as final products. Combining the general reaction pattern with theoretical calculations on geometry optimization at the M06-L/def2TZVP level suggests the formation of up to five Mo–S bonds together with Mo–C bonds, see Fig. 2d. This may be expected, since molybdenum is known to form a rich variety of sulfides [45]. However, the most intense final product is the charge transfer product SC_2_H_5_^+^. Other intense charge transfer products are S_2_C_2_H_6_^+^, S_3_C_3_H_9_^+^, SC_2_H_3_^+^ and S_2_C_3_H_7_^+^; their intensities are 88, 53, 41 and 20%, respectively, relative to SC_2_H_5_^+^ with 100%.

### Dimer Mo_2_^+^

**Fig. 4:**
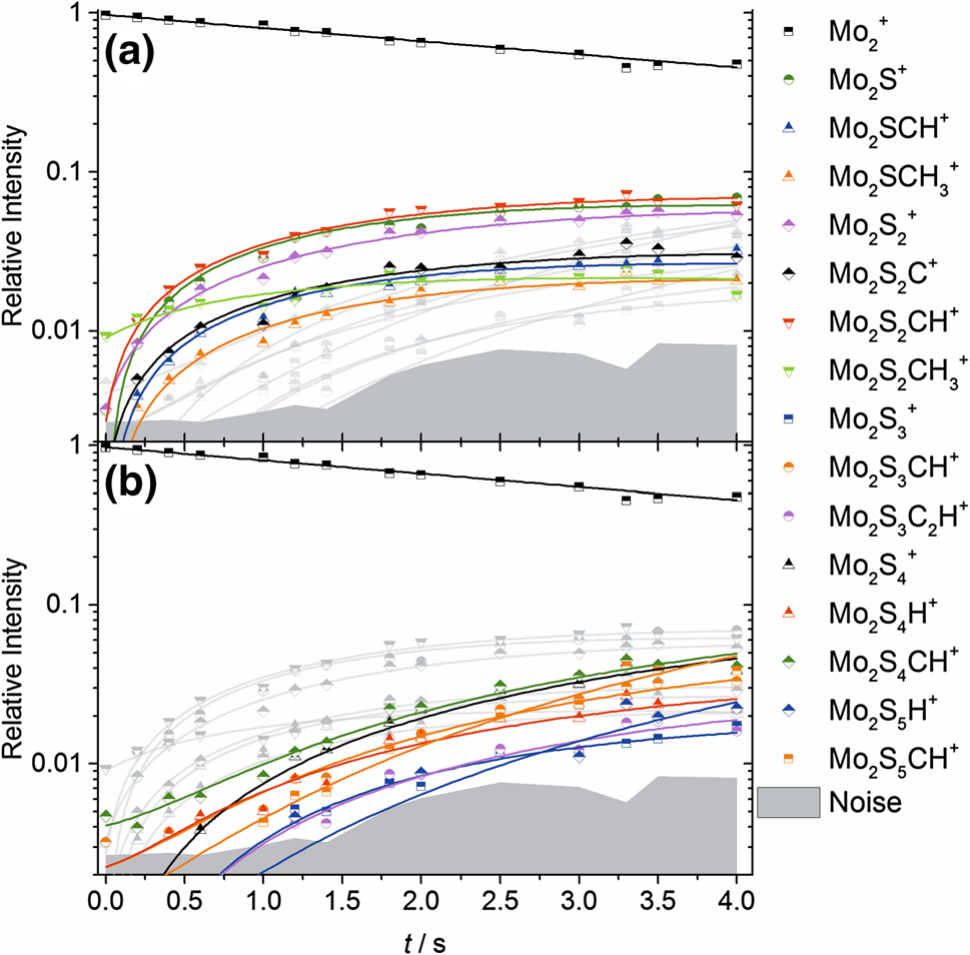
Kinetic fit of Mo_2_^+^ with DMDS during the first 4.0 s of the reaction at *p*_DMDS_ = 7.6(7) × 10^−9^ mbar. For the sake of clarity, the graph has been split in products containing up to two (**a**) or up to five (**b**) sulfur atoms respectively

Again, due to the vast variety of simultaneous reactions with different intermediates reacting through competing reaction channels, only the first 4.0 s of the reaction were analyzed with a kinetics fit. The kinetic model was developed iteratively by choosing stoichiometrically allowed and chemically reasonable reaction pathways and testing the quality of the fit. In Fig. 4 the time dependent relative intensities of the parent and product ions are shown. Due to the many intermediates which had to be taken into account, the graph is split in primary and higher order products. The primary reactions are listed in Table 2. A total of 46 different reaction channels were used in the kinetic model. At this high number of different reaction channels, the chosen model is not the only possibility, and secondary reactions cannot be assigned unambiguously. Therefore, only the first step, Mo_2_^+^ + CH_3_SSCH_3_, is discussed in more detail. The reactions 2.1, 2.4 and 2.6 are dominant, consistent with the tendency of molybdenum to directly extract sulfur from DMDS. All reactions included in Table 2 are again calculated to be exothermic.

The addition of CH_3_SSCH_3_ to Mo_2_^+^ is again considerably exothermic (up to − 5.9 eV, see Fig. 2b). DMDS tends to dissociate on the ion, forming a rhombic Mo_2_S_2_ core with CH_3_ groups attached to Mo atoms. The product ions form a high number of Mo–S and Mo–C bonds, similar to the case of Mo^+^. Predicted structural patterns are close to the ones calculated for neutral Mo_*x*_S_*y*_ gas phase compounds [18].

For reaction delays higher than 4.0 s, the acquired mass spectra were analyzed qualitatively, revealing that after a reaction time of approximately *t*_*r*_ = 19.0 s the dimer is completely converted to the products shown in Fig. S1. Clearly the most abundant products are Mo_2_S_6_C_3_H_9_^+^, Mo_2_S_7_C_3_H_9_^+^ and Mo_2_S_8_C_3_H_9_^+^. Again, no pure Mo_z_S_x_^+^ nor Mo_z_C_x_H_y_^+^ were found as final products. In the low mass region, charge transfer products were found. These were identified as SC_2_H_5_^+^, S_2_C_2_H_6_^+^ (ionized DMDS) and S_3_C_3_H_9_^+^; their intensities relative to the most intensive Mo_2_S_8_C_3_H_9_^+^ peak are 29, 19 and 11%, respectively.

### Trimer Mo_3_^+^

**Fig. 5:**
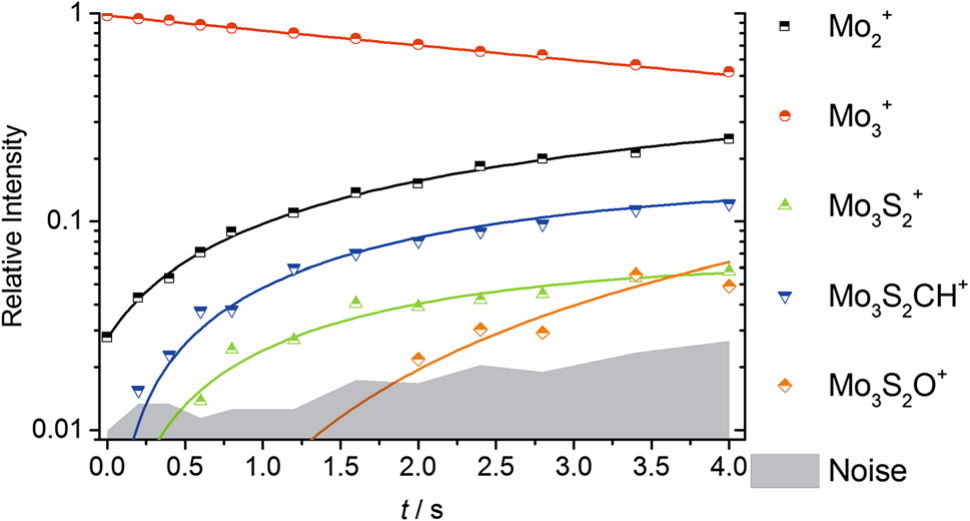
Kinetic fit of Mo_3_^+^ with DMDS during first 4.0 s of the reaction at *p*_DMDS_ = 6.8(5) × 10^−9^ mbar

For the first 4.0 s, the reaction proceeds smoothly, as shown in Fig. 5. Since Mo_2_^+^ is a product of the reaction, as shown in the mass spectrum in Fig S2, a large number of species can be observed as secondary products. Interestingly, two oxide species Mo_2_O^+^ and Mo_3_S_2_O^+^ are observed, suggesting that some of the species present might be capable of dehydrogenating water, which is likely to be present in traces in the vacuum system. The kinetic fit, Fig. 5, shows that the oxides are not primary products of Mo_3_^+^ + CH_3_SSCH_3_. The first step of the reaction kinetics was modelled as shown in Table 3. Calculated reaction energies are all exothermic.

Molybdenum trimer Mo_3_^+^ is predicted to be cyclic, in agreement with previous calculations on neutral systems [18]. The trimer unit is also kept during optimization for complexes with sulfur or carbon atoms, with the association product having a sandwich structure with a Mo_3_S_2_ core and two CH_3_ groups attached to Mo atoms, analogous to the association product of Mo_2_^+^ and DMDS (see Fig. 2c), with the reaction energy of − 7.0 eV. It is interesting to note that the only reaction that does not produce a Mo_3_-containing ion, reaction 3.1 resulting in Mo_2_^+^ + CH_3_Mo(S_2_)CH_3_, is with Δ*E* = − 0.98 eV only mildly exothermic, but has almost 50% branching ratio. This indicates that during the formation of the association product, the initial insertion of a Mo center into the S–S bond releases sufficient energy to liberate Mo_2_^+^.

Interestingly, no charge transfer products were found in the acquired mass spectra. This might be due to the relatively high noise level in the Mo_3_^+^ experiment, which leads to small intensities of secondary products of Mo_2_^+^ in the first 4 s of the reaction, below the noise level.

## Conclusions

Despite the vast variety of product ions forming, the early stage of each reaction was analyzed for all molybdenum cluster cations examined. Reasonable reaction schemes with realistic reaction rates in the order of 10^−10^ cm^3^ s^−1^ were found. Charge transfer reactions forming mixed S_x_C_y_H_z_^+^ clusters were observed only for the monomer and dimer in later stages of the reaction; in fact, charge transfer product ions became the dominant products in the monomer kinetics after longer times. At higher reaction delays, the dominant molybdenum containing product ions had the form Mo_*x*_S_*y*_(CH_3_)_*z*_^+^ with *x* = 1, 2, *y* > *x* and *z* = 2, 3. Supported by theoretical calculations, it can be assumed that these products consist of two or three methyl groups binding on the sulfided molybdenum cluster. The trimer was found to be relatively unstable, since a dissociative reaction channel was observed producing the fragment ion Mo_2_^+^ at a high rate of (5.3 ± 1.5) × 10^−10^ cm^3^ s^−1^. Albeit not being final products, pure Mo_x_S_y_^+^ product ions were observed for all examined species, illustrating the strong tendency of molybdenum to form sulfides by direct extraction of sulfur from DMDS.

In accordance with literature [16], the high sulfidation potential of DMDS can be confirmed. However, also a high yield of C_x_H_y_ species incorporated in the product clusters is observed. According to the calculated energetics from the literature [16], the binding energy of CH_y_ species incorporated in MoS_2_ increases from C via CH and CH_2_ to CH_3_. This trend is qualitatively reflected in the product distribution, where Mo_p_S_q_C_x_^+^ species are very rare.

## Electronic supplementary material

Below is the link to the electronic supplementary material.

Supplementary material 1 (DOCX 741 KB)
