# Supplementary material for: Gas-Phase Reactivity Studies of Small Molybdenum Cluster Ions with Dimethyl Disulfide
Source: Top Catal. 2017 Nov 27;61(1):20–7. doi: 10.1007/s11244-017-0864-3 (PMC6566215; doi:10.1007/s11244-017-0864-3)
Supplement: Supplementary file 1 — Supplementary material 1 (DOCX 741 KB) [file 11244_2017_864_MOESM1_ESM.docx]

Gas-Phase Reactivity Studies of Small Molybdenum Cluster Ions with Dimethyl Disulfide

Aristeidis Baloglou, Milan Ončák, Christian van der Linde, and Martin K. Beyer*

Institut für Ionenphysik und Angewandte Physik

Universität Innsbruck

Technikerstraße 25, 6020 Innsbruck, Austria

* Corresponding author: [martin.beyer@uibk.ac.at](mailto:martin.beyer@uibk.ac.at), +43-512-507 52680

Supporting Information

|  | | Mo^+^ | MoS^+^ | MoSCH^+^ | MoSCH_2_^+^ | MoSCH_3_^+^ | MoS_2_^+^ | MoS_2_C^+^ | MoS_2_CH^+^ | MoS_2_CH_2_^+^ | MoS_2_CH_3_^+^ | MoS_2_C_2_H_2_^+^ | MoS_3_^+^ | MoS_3_CH^+^ | MoS_3_C_2_H_3_^+^ |
| --- | --- | --- | --- | --- | --- | --- | --- | --- | --- | --- | --- | --- | --- | --- | --- |
| CH_3_SCH_2_^+^ |  | |  | 12.6 | 9.9 |  | 0.7 | 3.1 | 9.2 |  |  |  |  |  |  |
| CH_3_S_2_CH_3_^+^ | |  | 20.5 |  | 11.5 | 27.9 |  |  |  | 0.9 |  | 2.6 | 5.3 | 0.8 | 0.4 |
| MoS^+^ | | 1.6 |  |  |  |  |  |  |  |  |  |  |  |  |  |
| MoSCH^+^ | | 1.4 |  |  |  |  |  |  |  |  |  |  |  |  |  |
| MoSCH_2_^+^ | | 1.6 |  |  |  |  |  |  |  |  |  |  |  |  |  |
| MoSCH_3_^+^ | | 1.0 |  |  |  |  |  |  |  |  |  |  |  |  |  |
| MoS_2_^+^ | | 5.2 |  |  |  |  |  |  |  |  |  |  |  |  |  |
| MoS_2_C^+^ | | 1.1 |  |  |  |  |  |  |  |  |  |  |  |  |  |
| MoS_2_CH^+^ | | 4.3 |  |  |  |  |  |  |  |  |  |  |  |  |  |
| MoS_2_CH_2_^+^ | | 1.2 |  |  |  |  | 17.3 |  |  |  |  |  |  |  |  |
| MoS_2_CH_3_^+^ | | 2.6 |  |  |  |  |  |  |  |  |  |  |  |  |  |
| MoS_2_C_2_H_2_^+^ | | 1.8 |  |  |  |  |  |  |  |  |  |  |  |  |  |
| MoS_3_^+^ | |  |  |  |  |  |  |  |  | 17.2 |  | 11.9 |  |  |  |
| MoS_3_CH^+^ | |  |  |  |  |  |  |  |  | 17.1 |  | 4.0 |  |  |  |
| MoS_3_C_2_H_3_^+^ | | |  |  |  |  |  |  |  | 12.3 | 1.2 |  | 5.4 |  |  |
| MoS_4_CH^+^ | |  |  |  |  |  |  | 1.1 |  | 3.5 | 12.2 | 6.0 |  |  |  |
| MoS_4_C_2_H_3_^+^ | | |  |  |  |  |  | 1.6 | 4.2 | 4.5 |  | 4.3 |  |  |  |

**Tab. S1** Parameter matrix containing all calculated absolute reaction rates *k_abs_* (in 10^-10^ cm^3^/s) for the suggested reaction mechanism of Mo^+^ with DMDS, where each *k_ij_* entry means that the ion in the *j*-th column reacts towards the ion in the *i*-th row. The relative error of the listed values has been estimated to 28%.

**Tab. S2** Parameter matrix containing all calculated absolute reaction rates *k_abs_* (in 10^-10^ cm^3^/s) for the suggested reaction sequence of Mo_2_^+^ with DMDS, where each *k_ij_* entry means that the ion in the *j*-th column reacts towards the ion in the *i*-th row. The relative error of the listed values has been estimated to 29%.

|  | Mo_2_^+^ | Mo_2_S^+^ | Mo_2_SCH^+^ | Mo_2_SCH_3_^+^ | Mo_2_S_2_^+^ | Mo_2_S_2_C^+^ | Mo_2_S_2_CH^+^ | Mo_2_S_2_CH_3_^+^ | Mo_2_S_3_^+^ | Mo_2_S_3_CH^+^ | Mo_2_S_3_C_2_H^+^ | Mo_2_S_4_^+^ | Mo_2_S_4_H^+^ | Mo_2_S_4_CH^+^ |
| --- | --- | --- | --- | --- | --- | --- | --- | --- | --- | --- | --- | --- | --- | --- |
| Mo_2_S^+^ | 2.7 |  |  |  |  |  |  |  |  |  |  |  |  |  |
| Mo_2_SCH^+^ | 1.2 | 20.3 |  |  |  |  |  |  |  |  |  |  |  |  |
| Mo_2_SCH_3_^+^ | 0.8 | 0.1 | 17.9 |  |  |  |  |  |  |  |  |  |  |  |
| Mo_2_S_2_^+^ | 1.7 |  | 21.7 | 28.7 |  |  |  |  |  |  |  |  |  |  |
| Mo_2_S_2_C^+^ | 1.2 |  | 28.4 | 0.3 | 18.9 |  |  |  |  |  |  |  |  |  |
| Mo_2_S_2_CH^+^ | 2.6 |  |  | 10.4 | 8.1 | 39.2 |  |  |  |  |  |  |  |  |
| Mo_2_S_2_CH_3_^+^ | 1.4 |  |  |  |  |  | 14.6 |  |  |  |  |  |  |  |
| Mo_2_S_3_^+^ |  |  |  |  |  | 19.4 | 4.5 |  |  |  |  |  |  |  |
| Mo_2_S_3_CH^+^ |  |  |  |  |  | 3.6 | 2.9 | 15.7 | 40.4 |  |  |  |  |  |
| Mo_2_S_3_C_2_H^+^ |  |  |  |  |  | 3.4 | 7.0 |  | 8.6 | 22.3 |  |  |  |  |
| Mo_2_S_4_^+^ |  |  |  |  | 1.0 |  | 6.1 | 24.5 |  |  | 2.6 |  |  |  |
| Mo_2_S_4_H^+^ |  |  |  |  |  |  | 6.3 | 26.4 |  |  | 60.6 |  |  |  |
| Mo_2_S_4_CH^+^ |  |  |  |  | 4.0 | 9.6 | 1.4 | 13.7 |  |  |  | 5.7 | 19.3 |  |
| Mo_2_S_5_H^+^ |  |  |  |  |  |  |  |  |  |  |  |  | 17.8 | 5.5 |
| Mo_2_S_5_CH^+^ |  |  |  |  |  |  |  |  |  |  |  |  | 35.0 | 10.4 |

**Tab. S3** Parameter matrix containing all calculated absolute reaction rates *k_abs_* (in 10^-10^ cm^3^/s) for the suggested reaction sequence of Mo_3_^+^ with DMDS, where each *k_ij_* entry means that the ion in the *j*-th column reacts towards the ion in the *i*-th row. The relative error of the listed values has been estimated to 29%. The values denoted by * are not reliable due to unknown concentration of O_2_/H_2_O contamination.

|  | Mo_3_^+^ | Mo_3_S_2_^+^ | Mo_3_S_2_CH^+^ |
| --- | --- | --- | --- |
| Mo_2_^+^ | 5.3 |  |  |
| Mo_3_^+^ |  |  |  |
| Mo_3_S_2_^+^ | 2.0 |  |  |
| Mo_3_S_2_CH^+^ | 3.7 |  |  |
| Mo_3_S_2_O^+^ |  | 12.7* | 8.0* |

**
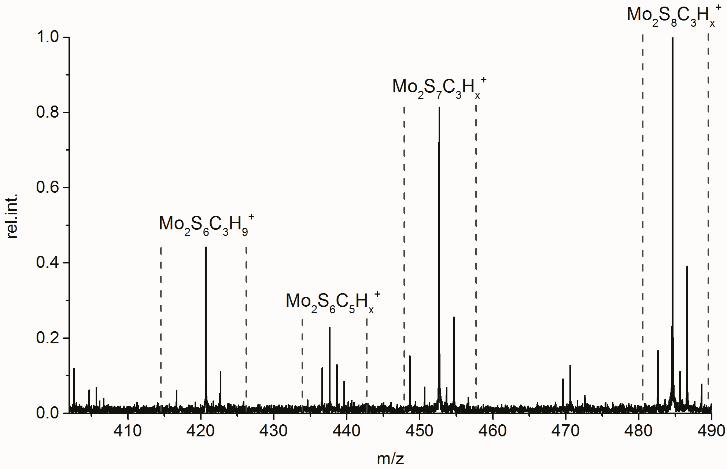
Mass spectra**

**Fig. S1** Mass spectrum of Mo_2_^+^ with DMDS after a reaction delay of 19.0 s showing the final molybdenum containing products of the reaction (*p*_DMDS_ = 7.6(7)×10^-9^ mbar). Intensities are shown relative to the most abundant product.


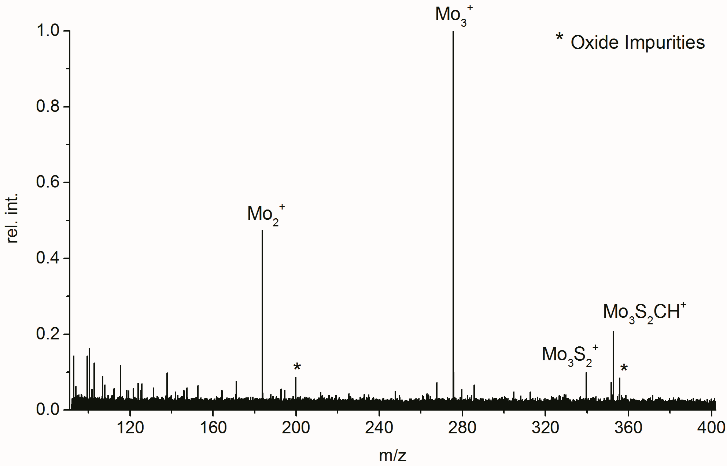


**Fig. S2** Mass spectrum of Mo_3_^+^ with DMDS after a reaction delay of 4.0 s (*p*_DMDS_ = 6.8(5)×10^‑9^ mbar).

**Benchmarking of *ab initio* methods**

Table S4 includes benchmarking of the computational approach using vertical and adiabatic ionization energies (VIE, AIE) of CH_3_SCH_3_, CH_3_SSCH_3_ and Mo.

For CH_3_SSCH_3_ and Mo, several methods were tested, showing that both M06-L/def2TZVP and B3LYP/def2TZVP methods reproduce reasonably both experimental results and results of higher-level methods. In the case of DMDS, AIE values found in the literature differ considerably; our results are in the best agreement with the value of Ref. 9 and 10 that predict about 8.2 eV. In the case of Mo, our results are close to the value reported in Ref. 12, 7.09 eV. Here, basis set deficiency is obviously compensated at the DFT level.

Finally, CH_3_SCH_3_ was included for completeness and because there is no controversy with respect to its adiabatic ionization energy value. Here, the B3LYP/def2TZVP method gives results slightly closer to the experimental values, with the M06-L/def2TZVP method underestimating both experimental VIE and AIE by about 0.1–0.2 eV.

Due to its performance for the VIE of Mo, we eventually picked the M06-L/def2TZVP method for all calculations presented in the main text.

**Tab. S4.** Vertical and adiabatic ionization energies (VIE and AIE, respectively; in eV) of CH_3_SCH_3_, CH_3_SSCH_3_ and Mo calculated at various levels of theory. Structures were optimized at the respective level unless stated otherwise.

| **atom/molecule** | **property** | **method** | **value** | **exp.** |
| --- | --- | --- | --- | --- |
| CH_3_SCH_3_ | VIE | M06-L/def2TZVP | 8.55 | 8.67-8.71 [1-3] |
|  |  | B3LYP/def2TZVP | 8.62 |  |
|  | AIE | M06-L/def2TZVP | 8.44 | 8.5-8.7 [2,4,5] |
|  |  | B3LYP/def2TZVP | 8.52 |  |
| CH_3_SSCH_3_ | VIE | M06-L/def2TZVP | 8.72 | 8.82-8.98 [6-8] |
|  |  | B3LYP/def2TZVP | 8.79 |  |
|  | AIE | M06-L/def2TZVP | 7.81 | 8.2±0.2;[9] 8.18±0.03;[10] 7.4±0.3 [11] |
|  |  | B3LYP/def2TZVP | 7.89 |  |
|  |  | M06/def2TZVP | 7.97 |  |
|  |  | BMK/def2TZVP | 8.01 |  |
|  |  | M06-L/aug-cc-pVDZ | 7.92 |  |
|  |  | M06-L/aug-cc-pVTZ | 7.81 |  |
|  |  | M06-L/aug-cc-pVQZ | 7.81 |  |
|  |  | MP2/def2TZVP | 7.96 |  |
|  |  | CCSD(T)/def2TZVP//MP2/def2TZVP | 7.94 |  |
| Mo | VIE | M06-L/def2TZVP | 7.01 | 7.09243±0.0004;[12] 7.22±0.06 [13] |
|  |  | M06-L/def2QZVPP | 7.00 |  |
|  |  | B3LYP/def2TZVP | 7.28 |  |
|  |  | M06/def2TZVP | 6.90 |  |
|  |  | MP2/def2TZVP | 6.68 |  |
|  |  | CCSD(T)/def2TZVP | 6.72 |  |
|  |  | CCSD(T)/aug-cc-pVTZ-PP | 6.89 |  |

**References**

[1] Chang, F.C.; Young, V.Y.; Prather, J.W.; Cheng, K.L., Study of methyl chalcogen compounds with ultraviolet photoelectron spectroscopy, *J. Electron Spectrosc. Relat. Phenom.*, **1986**, *40*, 363.

[2] Carnovale, F.; Livett, M.K.; Peel, J.B., Identification of the gas phase trimer (CH_3_)_2_S.(HF)_2_ by photoelectron spectroscopy, *J. Am. Chem. Soc.*, **1983**, *105*, 6788.

[3] Kobayashi, T., A new rule for photoelectron angular distributions of molecules, *Phys. Lett. A*, **1978**, *69*, 31.

[4] Kimura, K.; Katsumata, S.; Achiba, Y.; Yamazaki, T.; Iwata, S., Ionization energies, Ab initio assignments, and valence electronic structure for 200 molecules in Handbook of HeI Photoelectron Spectra of Fundamental Organic Compounds, Japan Scientific Soc. Press, Tokyo, **1981**.

[5] Aue, D.H.; Webb, H.M.; Davidson, W.R.; Vidal, M.; Bowers, M.T.; Goldwhite, H.; Vertal, L.E.; Douglas, J.E.; Kollman, P.A.; Kenyon, G.L., Proton affinities photoelectron spectra of three-membered-ring, *J. Am. Chem. Soc.*, **1980**, *102*, 5151.

[6] Baker, A.D.; Brisk, M.; Gellender, M., Photoelectron spectra and dihedral angles of disulfides, *J. Electron Spectrosc. Relat. Phenom.*, **1974**, *3*, 227.

[7] Kroto, H.W.; Suffolk, R.J., The photoelectron spectrum of an unstable species in the pyrolysis products of dimethyldisulphide, *Chem. Phys. Lett.*, **1972**, *15*, 545.

[8] Chang, F.C.; Young, V.Y.; Prather, J.W.; Cheng, K.L., Study of methyl chalcogen compounds with ultraviolet photoelectron spectroscopy, *J. Electron Spectrosc. Relat. Phenom.*, **1986**, *40*, 363.

[9] Leeck, D.T.; Kenttamaa, H.I., Heat of formation of the radical cation of dimethyl disulfide, *Org. Mass Spectrom.*, **1994**, *29*, 106.

[10] Li, W.-K.; Chiu, S.-W.; Ma, Z.-X.; Liao, C.L.; Ng, C.Y., Adiabatic ionization energy of CH_3_SSCH_3_, *J. Chem. Phys.*, **1993**, *99*, 8440.

[11] Butler, J.J.; Baer, T.; Evans, S.A., Jr., Energetics and structures of organosulfur ions: CH_3_SSCH_3_^+^, CH_3_SS^+^, C_2_H_5_S^+^, and CH_2_SH^+^, *J. Am. Chem. Soc.*, **1983**, *105*, 3451.

[12] Rayner, D.M.; Mitchell, S.A.; Bourne, O.L.; Hackett, P.A., First-ionization potential of niobium and molybdenum by double-resonance, field-ionization spectroscopy, *J. Opt. Soc. Am. B*, **1987**, *4*, 900.

[13] Rauh, E.G.; Ackermann, R.J., The first ionization potentials of the transition metals, *J. Chem. Phys.*, **1979**, *70*, 1004.

**Cartesian coordinates and energies of ions and molecules included in Tables 1-3 optimized at the M06-L/def2TZVP level of theory (in Ångstrom and a.u.), zero-point energy is included in all reported energies**

**Neutral molecules**

C2H6

-79.768678

C 0.000000 -0.756321 0.000000

C 0.000000 0.756321 0.000000

H -1.012784 -1.158910 0.000000

H 0.506543 -1.158658 0.877172

H 0.506543 -1.158658 -0.877172

H 1.012784 1.158910 0.000000

H -0.506543 1.158658 -0.877172

H -0.506543 1.158658 0.877172

CH3

-39.815556

C 0.000000 0.000000 0.000014

H 0.000000 1.077196 -0.000028

H -0.932879 -0.538598 -0.000028

H 0.932879 -0.538598 -0.000028

CH3SCH3

-477.960035

S 0.000000 0.656395 0.000023

C 1.367637 -0.507584 -0.000071

H 2.288222 0.070512 -0.000191

H 1.354577 -1.138129 -0.888462

H 1.354754 -1.138040 0.888385

C -1.367638 -0.507583 0.000035

H -1.354665 -1.138068 0.888470

H -2.288226 0.070508 0.000022

H -1.354663 -1.138102 -0.888376

CH3SH

-438.669424

S 0.047515 -0.661306 0.000000

C 0.047515 1.142841 0.000000

H 1.089988 1.449359 0.000000

H -0.426746 1.549174 0.889930

H -0.426746 1.549174 -0.889930

H -1.281832 -0.823851 0.000000

CH3SSCH3

-876.167555

S -0.447982 0.908669 -0.488691

S 0.447982 -0.908669 -0.488691

C 0.447982 1.828643 0.785042

C -0.447982 -1.828643 0.785042

H -0.029742 -2.834308 0.789634

H -0.307993 -1.387039 1.769117

H -1.507849 -1.883750 0.550049

H 0.029742 2.834308 0.789634

H 0.307993 1.387039 1.769117

H 1.507849 1.883750 0.550049

CH3S

-438.034004

S -0.686020 -0.000009 0.002088

C 1.092834 -0.000055 0.009255

H 1.411361 0.002498 -1.036418

H 1.503845 0.893844 0.475725

H 1.504105 -0.895864 0.471758

CH4

-40.480154

C 0.000000 -0.000045 -0.000061

H -0.886407 -0.557119 -0.287557

H 0.886776 -0.556575 -0.287471

H -0.000272 0.963772 -0.499797

H -0.000098 0.150194 1.075191

H2

-1.161720

H 0.000000 0.000000 0.371990

H 0.000000 0.000000 -0.371990

H

-0.503416

H 0.000000 0.000000 0.000000

Mo (S=7)

-68.112035

Mo 0.000000 0.000000 0.000000

SH

-398.738753

S 0.000000 0.000000 0.078993

H 0.000000 0.000000 -1.263892

Mo.CH3SSCH3 (S=3)

-944.442170

Mo 0.486886 0.000006 -0.000310

S -1.576992 0.001110 1.035566

S -1.577656 -0.000676 -1.035074

C 1.598518 -1.768600 0.001117

H 2.232505 -1.805997 0.893792

H 2.232116 -1.806821 -0.891837

H 0.950703 -2.646212 0.001666

C 1.599842 1.767833 -0.000700

H 2.233697 1.806214 -0.893397

H 0.952376 2.645709 0.000200

H 2.233604 1.804521 0.892210

SSH

-796.969781

S 0.040117 -1.014882 0.000000

S 0.040117 0.937761 0.000000

H -1.283748 1.233950 0.000000

**Cations**

CH3SSCH3+

-875.880518

S -0.596829 0.797644 -0.000002

S 0.596829 -0.797644 -0.000002

C 0.596829 2.134011 0.000004

C -0.596829 -2.134011 0.000004

H 0.002273 -3.042329 -0.000032

H -1.210138 -2.095490 0.897178

H -1.210193 -2.095455 -0.897131

H -0.002273 3.042329 -0.000032

H 1.210138 2.095490 0.897178

H 1.210193 2.095455 -0.897131

Mo+ (S=6)

-67.854422

Mo 0.000000 0.000000 0.000000

MoS2C2H2+ (S=2)

-941.767144

S -1.067481 1.241615 0.043924

Mo 0.423250 -0.401470 -0.209490

S -1.919954 -0.551549 0.216759

C 2.177103 -0.141303 0.689874

H 2.902200 -0.545295 1.385437

C 1.949046 0.893258 -0.088729

H 2.363373 1.854240 -0.364672

MoS2CH2+ (S=2)

-903.686859

S 1.679732 0.503208 -0.546994

Mo -0.205391 -0.563471 -0.024460

S -1.784045 0.770603 -0.034908

C 1.242661 0.381646 1.152816

H 1.025552 1.317145 1.657982

H 1.813914 -0.322228 1.762879

MoS2CH3+ (S=1)

-904.280292

Mo 0.100419 0.257876 -0.361496

S -1.875517 -0.689827 0.247531

S 1.614748 -0.976196 0.306408

C 0.330658 1.795311 0.650870

H 1.069577 2.176320 1.348878

H -0.550604 2.440686 0.509540

H -2.548246 0.436709 0.556146

MoS2CH+ (S=1)

-903.067803

C -0.945595 0.841563 0.838556

S -1.915343 0.272905 -0.424725

S 1.880542 0.617589 -0.255713

Mo 0.172215 -0.502446 0.107549

H -1.002663 1.805443 1.338595

MoS2C+ (S=4)

-902.453301

S 1.593734 0.932535 -0.000029

Mo 0.001453 -0.871355 0.000002

S -1.596851 0.928136 -0.000029

C -0.001861 1.137692 0.000139

MoS2+ (S=2)

-864.378416

Mo 0.000000 0.000000 0.539793

S 0.000000 -1.655418 -0.708478

S 0.000000 1.655418 -0.708478

MoSCH2+ (S=4)

-505.410777

C -1.003934 1.158563 -0.000059

S -1.395260 -0.553469 0.000020

Mo 0.735022 -0.035474 -0.000013

H -1.260922 1.696796 0.910934

H -1.262257 1.697251 -0.910355

MoSCH3+ (S=3)

-506.025581

C 1.590784 1.106376 0.000067

Mo 0.242145 -0.443613 0.000011

S -1.584579 0.501755 0.000006

H 1.560913 1.725689 0.896647

H 1.556416 1.728560 -0.894360

H 2.521133 0.511153 -0.003253

MoSCH+ (S=5)

-504.802612

Mo 0.000000 0.765166 0.000000

S -0.500578 -1.561192 0.000000

C 1.007513 -0.948055 0.000000

H 1.964164 -1.469565 0.000000

MoS+ (S=4)

-466.109770

Mo 0.000000 0.000000 0.568530

S 0.000000 0.000000 -1.492391

Mo2+ (S=2)

-136.122950

Mo 0.000000 0.000000 0.968894

Mo 0.000000 0.000000 -0.968894

Mo2S2CH3+ (S=1)

-972.604736

Mo -0.912838 -0.000281 -0.329191

Mo 0.946016 0.000243 0.697683

S 0.700969 1.700909 -0.763308

S 0.701860 -1.700770 -0.762998

C -2.533474 -0.000140 0.950446

H -3.078217 -0.893167 0.604760

H -2.481748 0.000849 2.033583

H -3.077913 0.892526 0.603183

Mo2S2CH+ (S=3)

-971.352070

Mo -0.896095 -0.971369 -0.103298

Mo 0.719401 0.619402 -0.331156

S -1.647067 1.239389 0.120702

S 2.351139 -0.547025 0.393365

C -0.549897 0.506609 1.278436

H -0.544622 0.665142 2.351407

Mo2S2C+ (S=4)

-970.752585

Mo 0.754343 0.638516 -0.467626

Mo -1.296164 -0.653165 -0.114495

S 2.119117 -0.929433 0.604194

S -0.900245 1.415525 0.863041

C 0.542421 -1.193702 0.162224

Mo2S2+ (S=2)

-932.689286

S 1.951295 -0.047290 -0.000065

Mo -0.000163 1.187679 0.000023

S -1.951335 -0.047826 -0.000065

Mo 0.000179 -1.151444 0.000026

Mo2SCH3+ (S=1)

-574.307766

S -1.129355 1.446416 0.364862

Mo 0.864212 0.325274 -0.294021

Mo -0.961109 -0.726548 -0.010318

C 2.347551 -0.650091 0.754174

H 2.953237 0.055252 1.329136

H 2.232551 -1.580406 1.300385

H 2.868240 -0.863426 -0.210127

Mo2SCH+ (S=3)

-573.101942

Mo -1.117577 -0.214102 -0.152531

Mo 1.117613 -0.214289 -0.152470

S 0.000167 1.736675 0.312623

C -0.000881 -1.280459 1.014038

H 0.001098 -2.111626 1.723807

Mo2S+ (S=2)

-534.407675

Mo -0.638689 -0.938903 0.000000

Mo 0.000000 1.075565 0.000000

S 1.676559 -0.358737 0.000000

Mo3+ (S=2)

-204.361543

Mo 1.154955 -0.634139 0.000000

Mo 0.000000 1.268533 0.000000

Mo -1.154955 -0.634394 0.000000

Mo3S2CH+ (S=3)

-1039.699544

S 2.132498 0.135762 -0.731928

Mo 0.649696 -1.159036 0.396747

Mo 0.339633 1.381398 -0.046886

Mo -1.437020 -0.074373 -0.518760

S -0.751488 0.250562 1.694887

C -0.475280 -1.679910 -1.060733

H -0.441470 -2.317234 -1.949196

Mo3S2+ (S=2)

-1000.989230

S 1.022777 -0.000770 -1.602671

Mo -0.401759 -1.263017 -0.171164

Mo -0.400854 1.263105 -0.171593

S -2.180298 0.000814 0.535600

Mo 1.243573 -0.000105 0.749261

MoS4C3H9+ (S=3)

-1780.559973

Mo -0.159013 -0.129920 -0.028110

S 1.223218 1.406822 1.000925

S 2.058137 0.750849 -0.738850

S -0.608755 -2.104103 0.974214

S -2.012953 0.885507 -0.803432

C -2.645064 2.080913 0.414612

H -3.596808 2.438440 0.029467

H -2.801886 1.597986 1.374564

H -1.952652 2.912455 0.511062

C 3.247508 -0.541181 -0.291022

H 3.487743 -1.065143 -1.214934

H 4.141172 -0.060071 0.098598

H 2.833031 -1.226457 0.442804

C -1.038572 -2.571452 -0.734342

H -0.457145 -3.438189 -1.035440

H -2.106009 -2.760256 -0.810064

H -0.806491 -1.777036 -1.476656

MoS4C3H9+ (S=3)

-1780.557096

Mo 0.002301 0.384577 -0.042493

S -1.420590 -0.806522 -1.413376

S -2.500556 0.129170 0.049493

S 1.455741 -0.388717 1.565296

S 2.502445 0.135732 -0.111563

C 2.864282 -1.411626 -0.975993

H 3.655501 -1.931807 -0.443054

H 1.980702 -2.037707 -1.058290

H 3.218828 -1.124867 -1.964846

C -2.856627 -1.135474 1.293257

H -3.201622 -0.598874 2.175788

H -3.653525 -1.774745 0.922412

H -1.972978 -1.721632 1.528234

C -0.072725 2.458642 -0.277610

H 0.919147 2.906671 -0.340593

H -0.592571 2.618099 -1.229222

H -0.652371 2.928798 0.518757

MoS4C3H9+ (S=1)

-1780.563323

C 1.482479 1.910450 -0.784166

Mo 0.376244 0.296633 0.181506

C 0.620657 1.074402 2.081597

S 0.668117 -1.968540 0.139008

S 2.172393 -0.861518 -0.617901

S -1.381602 1.208689 -0.985076

S -2.149210 -0.008208 0.459547

H 1.035196 2.805100 -0.344650

H 2.540049 1.876490 -0.533545

H 1.377027 1.956017 -1.865039

H 0.173302 0.402428 2.816470

H 1.685159 1.211401 2.279745

H 0.114342 2.043295 2.113290

C -2.663029 -1.522054 -0.375089

H -1.906323 -1.859298 -1.079352

H -2.810471 -2.265866 0.406519

H -3.606352 -1.331696 -0.880012

MoS5C2H6+ (S=2)

-2138.932940

Mo 0.018659 0.488524 0.100400

S -0.559529 2.317926 -0.719136

S -1.526812 -0.405414 1.581359

S -1.599037 -1.381566 -0.206236

C -3.015342 -0.719974 -1.122222

H -3.119524 0.346795 -0.955560

H -3.902260 -1.255915 -0.794328

H -2.825864 -0.926189 -2.174007

S 2.220911 0.512826 0.826106

S 2.033369 -0.576579 -0.886068

C 2.004899 -2.313523 -0.370937

H 3.026678 -2.620367 -0.162679

H 1.381251 -2.456027 0.507628

H 1.616261 -2.880422 -1.215288

MoS5C2H6+ (S=2)

-2138.915842

Mo 0.191645 -0.044129 0.275024

S -2.058760 -0.882190 -0.585261

S -1.736369 -0.496723 1.400450

C -2.990515 0.530347 -1.205231

H -2.543420 1.467219 -0.881202

H -2.958498 0.452880 -2.291149

H -4.019580 0.448557 -0.865330

S 1.462187 -1.851067 -0.189600

C 3.038891 -1.230169 -0.857398

H 3.646253 -2.108829 -1.059725

H 2.865654 -0.681726 -1.778857

H 3.526571 -0.598655 -0.119497

S 0.367872 1.889700 -0.948157

S 1.411549 1.782338 0.811351

MoS5C2H6+ (S=2)

-2138.919394

C 0.934245 2.145937 -0.810332

Mo 0.245147 0.252693 0.061425

S -0.058035 0.810449 2.045180

S -1.848707 -1.013683 -0.488894

S -1.528296 0.785907 -1.354970

S 1.428237 -1.839443 -0.131954

S 2.580450 -0.260379 -0.262474

C -3.060446 -0.743545 0.830391

H -4.047646 -0.879997 0.396100

H -2.881981 -1.502572 1.589041

H -2.953304 0.251189 1.250338

H 0.158077 2.901235 -0.719401

H 1.818875 2.468887 -0.266147

H 1.188645 2.008196 -1.860344

MoS5C3H9+ (S=1)

-2178.823581

C 0.468181 2.404684 -0.119561

Mo 0.005327 0.292079 0.072149

S -2.084723 -0.861030 -0.680671

C -3.012937 -1.393554 0.780576

S 1.134759 -1.244088 -1.298461

S 2.443808 0.118478 -0.573728

C 3.360655 -0.720343 0.743627

S -2.038945 1.160896 -0.635557

H -4.057536 -1.472061 0.489295

H -2.633316 -2.373158 1.063808

H -2.887072 -0.684254 1.592393

H 3.730056 0.058631 1.408390

H 2.720142 -1.410381 1.282762

H 4.197650 -1.239920 0.284104

H -0.329052 3.080155 0.176825

H 1.353632 2.604589 0.481144

H 0.709831 2.576759 -1.171078

S 0.049885 -0.121279 2.121806

MoS5C3H9+ (S=3)

-2178.802974

C -2.848006 -1.577401 -1.137109

S -2.443048 -0.033642 -0.284658

Mo 0.020688 -0.028149 0.154045

S -1.735584 -0.567927 1.557412

S 0.602519 2.151529 -0.003149

C -0.722390 3.176796 -0.706583

S 2.308605 -0.950709 0.557009

S 1.240531 -1.566899 -1.076616

C 3.551697 0.201682 -0.071258

H -2.059343 -2.313740 -1.013303

H -3.788842 -1.953447 -0.744498

H -2.967730 -1.320253 -2.188445

H 3.917864 0.750336 0.795178

H 4.361605 -0.375740 -0.508485

H 3.119989 0.885551 -0.795956

H -1.045307 2.784479 -1.666270

H -1.557214 3.229595 -0.013228

H -0.306095 4.171397 -0.845153
